# The biological effects and thermal degradation of NPB-22, a synthetic cannabinoid

**DOI:** 10.1007/s11419-023-00679-5

**Published:** 2024-01-31

**Authors:** Akihiro Takeda, Takahiro Doi, Akiko Asada, Katsuhiro Yuzawa, Akemichi Nagasawa, Kai Igarashi, Tomokazu Maeno, Atsuko Suzuki, Seiko Shimizu, Nozomi Uemura, Jun’ichi Nakajima, Toshinari Suzuki, Akiko Inomata, Takaomi Tagami

**Affiliations:** 1 Division of Hygienic Chemistry, Osaka Institute of Public Health, 1-3-3 Nakamichi, Higashinari-ku, Osaka, 537-0025 Japan; 2 Department of Pharmaceutical and Environmental Sciences, Tokyo Metropolitan Institute of Public Health, 3-24-1 Hyakunin-cho, Shinjuku-ku, Tokyo, 169-0073 Japan; 3 Tolyo Metropolitan Island Public Health Center, 2466-2 Okago, Hachijo-machi, Hachijojima, Tokyo, 100-1492 Japan

**Keywords:** NPS, Synthetic cannabinoid, NPB-22, Inhalation exposure test, Thermal degradation

## Abstract

**Purpose:**

NPB-22 (quinolin-8-yl 1-pentyl-1*H*-indazole-3-carboxylate), Adamantyl-THPINACA (*N*-(1-adamantantyl)-1-[(tetrahydro-2*H*-pyran-4-yl)methyl]-1*H*-indazole-3-carboxamide), and CUMYL-4CN-B7AICA (1-(4-cyanobutyl)-*N*-(2-phenylpropan-2-yl)-1*H*- pyrrolo[2,3-b]pyridine-3-carboxamide), synthetic cannabinoids were evaluated in terms of CB_1_ (cannabinoid receptor type 1) and CB_2_ (cannabinoid receptor type 2) activities, and their biological effects when inhaled similar to cigarettes were examined.

**Methods:**

The half maximal effective concentration values of the aforementioned synthetic cannabinoids at the CB_1_ and CB_2_ were investigated using [^35^S]guanosine-5’-*O*-(3-thio)-triphosphate binding assays. In addition, their biological effects were evaluated using the inhalation exposure test with mice. The smoke generated was recovered by organic solvents in the midget impingers, and the thermal degradation compounds of the smoke components were identified and quantified using a liquid chromatography–photo diode array detector.

**Results:**

NPB-22 and Adamantyl-THPINACA had equivalent CB_1_ activity in in vitro assays. Meanwhile, NPB-22 had a weaker biological effect on some items on the inhalation exposure test than Adamantyl-THPINACA. When analyzing organic solvents in the midget impingers, it was revealed that NPB-22 was degraded to 8-quinolinol and pentyl indazole 3-carboxylic acid by combustion. In addition, these degradation compounds did not have CB_1_ activity.

**Conclusion:**

It was estimated that the biological effects of NPB-22 on the inhalation exposure test weakened because it underwent thermal degradation by combustion, and the resultant degradation compounds did not have any CB_1_ activity in vitro.

**Supplementary Information:**

The online version contains supplementary material available at 10.1007/s11419-023-00679-5.

## Introduction

Synthetic cannabinoids (SCs) have been originally developed based on the chemical structure of tetrahydrocannabinol (THC) contained in cannabis to study the endocannabinoid system [1]. They are functionally similar to THC, bind to cannabinoid receptor type 1 (CB_1_) and cannabinoid receptor type 2 (CB_2_), and are expected to have cannabis-like effects. They were mixed with or sprayed on plant materials and sold as herbs; SCs were used to obtain cannabis-like effects around the mid-2000s.

The governments of various nations have regulated the amount of SCs detected in products; however, illicit manufacturers have synthesized new SCs by replacing a part of the chemical structure of SCs distributed previously in drug markets to circumvent the regulation. NPB-22 (quinolin-8-yl 1-pentyl-1*H*-indazole-3-carboxylate), an indazole analog of PB-22 (quinolin-8-yl 1-pentyl-1*H*-indole-3-carboxylate), was first found in Hungary in January 2014 [2] and in Ankara, Turkey, in the same year [3]. Moreover, it was detected in authentic blood samples obtained in cases of driving under the influence of drugs between January 2013 and November 2015 in Germany [4]. These new SCs have not been adequately researched on in terms of their biological effects, and NPB-22 has not been thoroughly examined. Accordingly, we evaluated the CB_1_ and CB_2_ activities of NPB-22 using [^35^S]guanosine-5’-*O*-(3-thio)-triphosphate ([^35^S]GTPγS) binding assays. Furthermore, because the abusers of SCs have created handmade cigarettes by rolling the plant material in cigarette paper and inhaled the smoke from ignited cigarettes to absorb SCs [5–7], we have also evaluated the biological effects of these new SCs using the inhalation exposure test in which mice are exposed to smoke from burning plants sprayed with a solution containing SCs similar to cigarettes [8].

In this study, it was revealed that NPB-22 possessed a strong binding activity on the same level with the positive control, CP-55,940 (2-((1*R*,2*R*,5*R*)-5-hydroxy-2-(3-hydroxypropyl)cyclohexyl)-5-(2-methyloctan-2-yl)phenol), and was expected to exhibit cannabis-like effects. However, the biological effects of NPB-22 were weaker than expected. Accordingly, the smoke rising from plants sprayed with NPB-22 burned in the inhalation exposure test was collected using midget impingers and was analyzed by liquid chromatography. The results of the analysis showed the degradation of NPB-22 by combustion. Conventional cigarettes are directly ignited, the temperature of the combustion point reaches 800–900 °C, and the thermal degradation of SCs has been reported [9, 10]. In this paper, we report the binding activity on cannabinoid receptors and the biological effects of NPB-22 and its degradation by combustion.

## Materials and methods

### Reagents

Methyl 1*H*-indazole-3-carboxylate (CAS No. 43120-28-1) was purchased from Accela ChemBio Co., Ltd. (SanDiego, CA, USA). 4-(Iodomethyl)tetrahydro-2*H*-pyran (CAS No. 101691–94-5) was purchased from Acros Organics (Antwerp, Belgium). 1*H*-Pyrrolo[2,3-*b*]pyridine-3-carboxylic acid (CAS No. 156270-06-3) was purchased from Combi-Blocks Inc. (SanDiego, CA, USA). 1-Bromopentane (CAS No. 110-53-2) was purchased from FUJIFILM Wako Pure Chemical Corporation (Osaka, Japan). 8-Quinolinol (CAS No. 148-24-3) was purchased from Nacalai Tesque, Inc. (Kyoto, Japan). 5-Bromovaleronitrile (CAS No. 5414-21-1), potassium *tert*-butoxide (CAS No. 865-47-4), 1-adamantanamine hydrochloride (CAS No. 768-94-5), and cumylamine (CAS No. 585-32-0) were purchased from Tokyo Chemical Industry Co., Ltd. (Tokyo, Japan). All other reagents used in this study were purchased from FUJIFILM Wako Pure Chemical Corporation (Osaka, Japan). Purified water was obtained from tap water using Elix Advantage-10 from Merck Millipore (Burlington, MA, USA).

### Chemical synthesis

**Fig. 1:**
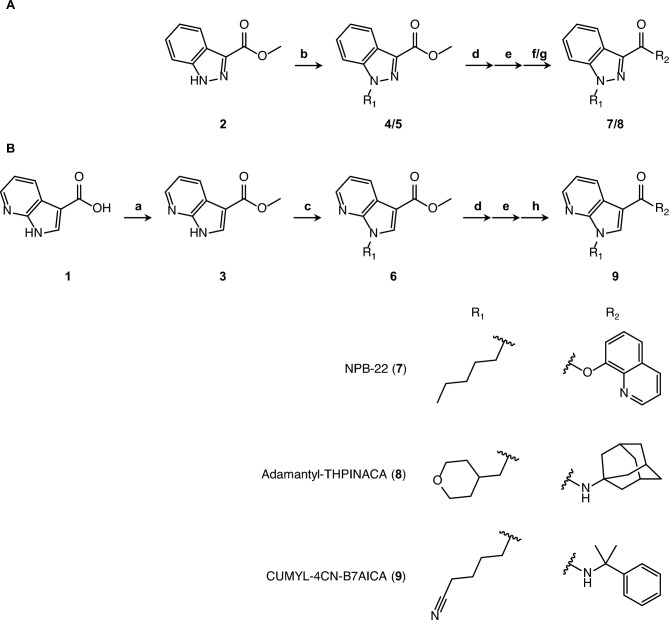
Synthesis route for the compounds under study

The synthetic pathways of NPB-22, Adamantyl-THPINACA (*N*-(1-adamantanyl)-1-[(tetrahydro-2*H*-pyran-4-yl)methyl]-1*H*-indazole-3-carboxamide), and CUMYL-4CN-B7AICA (1-(4-cyanobutyl)-*N*-(2-phenylpropan-2-yl)-1*H*- pyrrolo[2,3-b]pyridine-3-carboxamide) are shown in Fig. 1.

NPB-22 required methyl 1*H*-indazole-3-carboxylate (compound **2**) as a starting material. Compound **2** was *N*-alkylated by 1-bromopentane as alkyl halide under basic conditions to obtain compound **4** through the synthetic pathway **b**. Subsequently, NPB-22 (compound **7**) was yielded via the deprotection of compound **4** by hydrolysis of the methyl group (pathway **d**), chlorination (pathway **e**), and esterification with 8-quinolinol (pathway **f**).

Adamantyl-THPINACA was synthesized according to a previously reported method [11]. Specifically, Adamantyl-THPINACA also required methyl 1*H*-indazole-3-carboxylate (compound **2**) as a starting material. Compound **2** was *N*-alkylated by 4-(iodomethyl) tetrahydro-2*H*-pyran as alkyl halide under basic conditions to obtain compound **5** through synthetic pathway **c**. Subsequently, Adamantyl-THPINACA (compound **8**) was yielded by deprotecting compound **5** via hydrolysis of the methyl group (pathway **d**), chlorination (pathway **e**), and amidation with 1-adamantanamide hydrochloride (pathway **g)**.

CUMYL-4CN-B7AICA required 1*H*-pyrrolo[2, 3-*b*]pyridine-3-carboxylic acid (compound **1**) as a starting material. Compound **1** was protected by a hydroxy group of carboxylic acids, and compound **3** was obtained (pathway **a**). Compound **3** was *N*-alkylated by 5-bromovaleronitrile as alkyl halide under basic conditions to obtain compound **6** through synthetic pathway **c**. Subsequently, CUMYL-4CN-B7AICA (compound **9**) was yielded by deprotecting compound **6** via hydrolysis of the methyl group (pathway **d**), chlorination (pathway **e**), and amidation with cumylamine (pathway **h)**. NPB-22 and CUMYL-4CN-B7AICA are described in the Supplementary Material.

### In vitro assays to evaluate CB_1_ and CB_2_ activities

CB_1_/CB_2_ activities were evaluated using [^35^S]Guanosine-5’-*O*-(3-thio)-triphosphate ([^35^S]GTPγS) binding assays performed at ADME and Tox. Research Institute, Sekisui Medical Co., Ltd. (Tokai-mura, Ibaraki, Japan). The assay conditions were as previously described in [8, 12], except for the tested concentration levels of the compounds ranging from 1 × 10^−12^ to 1 × 10^−5^ M.

### Inhalation exposure test

An inhalation exposure test was performed to examine the biological effects of the test compounds at the Tokyo Metropolitan Institute of Public Health (Tokyo, Japan). We referred to our previous study [8] for the results from marshmallow (*Althaea officinalis*) as the negative control. Refer to our previous study [8] for the conditions and evaluation methods.

### Standard preparation for liquid chromatography methods

Standard stock solutions were prepared at a concentration of approximately 100 μg/mL in acetonitrile and stored at − 20 °C until use. When necessary, the standard stock solutions were diluted with 0.1% aqueous formic acid for high-performance liquid chromatography analysis with a photodiode array detector (HPLC–PDA).

### Proposed method for HPLC–PDA

HPLC–PDA was performed using LC-2040C 3D Plus (Shimadzu, Kyoto, Japan). For chromatographic separation, XSELECT HSS T3 (3.5 μm particle size, 150 × 4.6 mm i.d.) (Waters, Milford MA, USA) was used. The column temperature was maintained at 40 °C. The mobile phase was composed of solvent A (0.1% aqueous formic acid solution) and solvent B (0.1% formic acid acetonitrile solution). The flow rate of the mobile phase was 1.0 mL/min, and the injection volume was 10 μL. The gradient condition was as follows: initially 99%A/1%B (0–1.50 min), linearly changed to 10%A/90%B (1.50–20.55 min), and then linearly changed to 1%A/99%B (20.55–26.55 min). The wavelength of the ultraviolet (UV) spectra was 200–400 nm.

## Results

### ***Functional activity at the CB***_***1***_*** and CB***_***2***_

Table 1 shows the results of the [^35^S]GTPγS binding assays of NPB-22, Adamantyl-THPINACA, CUMYL-4CN-B7AICA, 8-quinolinol, and pentyl indazole 3-carboxylic acid. NPB-22 possessed more affinity to CB_1_ than CB_2_. The CB_1_ agonist activity of NPB-22 was equivalent to that of Adamantyl-THPINACA and stronger than that of CUMYL-4CN-B7AICA. Adamantyl-THPINACA did not possess CB_2_ agonist activity, and NPB-22 possessed stronger CB_2_ agonist activity than CUMYL-4CN-B7AICA. 8-quinolinol and pentyl indazole 3-carboxylic acid did not possess both CB_1_, and CB_2_ agonist activities.

**Table 1:** Activities of test compounds at human CB_1_ and CB_2_

Compound name	EC_50_ (mol/L)	
	CB_1_	CB_2_
NPB-22	4.97 × 10^–9^	2.69 × 10^–8^
Adamantyl-THPINACA	3.30 × 10^–9^	> 1 × 10^–5^
CUMYL-4CN-B7AICA	4.26 × 10^–8^	8.60 × 10^–7^
8-Quinolinol	> 1 × 10^–5^	> 1 × 10^–5^
1-Pentyl-1*H* -indazole-3-carboxylic acid	> 1 × 10^–5^	> 1 × 10^–5^
CP-55,940^a^	1.73 × 10^–9^	9.99 × 10^–10^

^a^*n* = 8 average

### Observation of the biological effects

The results of the inhalation exposure test of NPB-22, Adamantyl-THPINACA, and CUMYL-4CN-B7AICA are shown in Table 2. Regarding general behavior, aggressiveness, passivity, stereotype, grooming, vocalization, sound response, touch response, pain response, and verticalness of all subjects treated with the compounds under study were observed. In terms of neurological behavior, the spontaneous activity, abnormal gait, abnormal position, muscle tone, Straub tail reaction, righting reflex, pinna reflex, corneal reflex, tendon reflex, tremor, convulsion, grip strength, and detached finger of all subjects treated with the compounds under study were observed. Regarding autonomic behavior, exophthalmos, pupil size, palpebral opening, shed tears, salivation, respiratory rate, heart rate, piloerection, temperature, and skin color were observed.

**Table 2:** Mean values of the score in general, neurological, and anatomical behavior (*n* = 5)

Observation time (h)	Marshmallow^a^			NPB-22			Adamantyl-THPINACA			CUMYL-4CN-B7AICA		
	0.25	0.5	1	0.25	0.5	1	0.25	0.5	1	0.25	0.5	1
General behavior												
Agressiveness	0	0	0	0	0.4	0.8	1.4	0.8	0.8	0.4	0.4	0.4
Passivity	0	− 0.6	− 0.6	− 0.4	0	0	− 0.4	− 0.4	0.0	− 0.4	− 0.4	− 0.4
Stereotype	0.2	0	0	0	0	0.2	0	0	0	0	0	0
Grooming	− 2.8	− 1.4	− 0.4	− 2	− 1.2	− 1.6	− 3	− 3	− 2.4	− 1	− 0.8	− 0.4
Vocalization	0	0	0	0	0	0	0	0	0	0	0	0
Sound response	0	0.2	0	− 0.2	0.2	0.2	− 1	− 1	0.2	0	0.2	0.2
Touch response	0	− 0.2	− 0.4	− 0.8	0.2	0.6	0.2	0	0.4	− 0.2	0	− 0.2
Pain response	− 0.4	− 0.2	− 0.2	− 0.6	0.2	0.6	− 0.2	1	0.8	0	0.2	0
Verticalness	− 0.6	− 1.6	− 0.8	− 2	− 1.6	− 0.8	− 3	− 3	− 1.4	0	− 0.6	− 0.4
Neurological behavior												
Spontaneous activity	− 0.8	− 0.2	− 0.2	− 1.8	− 0.8	− 1	− 2	− 1.2	− 0.6	− 0.2	− 0.4	− 0.4
Abnormal gait	0	0	0	− 2	− 1.4	− 0.8	− 2.4	− 1.2	− 0.6	− 0.6	− 0.2	− 0.4
Abnormal position	0	0	0	− 1.6	− 1.2	− 0.8	− 2.4	− 1.2	− 1	− 1.2	− 0.4	− 0.6
Muscle tone	0	0	0	− 1.8	0	− 0.4	1.6	1.4	0	− 0.2	− 0.6	− 0.4
Straub tail reaction	0	0	0	0	0	0	1	0	0	0	0	0
Righting reflex	0	0	0	0	0	0	− 0.4	0	0	0	0	0
Pinna reflex	0	− 0.2	− 0.4	0.2	0.2	0.6	0.0	0.6	0.4	− 0.2	0.2	0.4
Corneal reflex	0	0	0	0.2	0.2	0.6	0.0	0.6	0.4	− 0.4	0.2	0.4
Tendon reflex	0	− 0.6	0.2	− 0.2	− 0.2	0.4	0.2	0.6	0.4	− 0.4	− 0.2	0.2
Tremor	0	0	0	0	0.4	0.8	0	0	0	0.8	0.4	0.6
Convulsion	0	0	0	0.2	0	0	0	0	0	0	0	0
Grip strength	0	0.4	0	− 0.2	0	0	− 1.6	− 0.8	− 0.2	0.2	0.2	0.4
Detached finger	0	0	0	0	0	0	0	0	0	0	0	0
Autonomic behavior												
Exophthalmos	0	0	0	0	0	0	0	0	0	0	0	0
Pupil size	0	0	− 0.2	0.6	0.4	0.4	0.2	0.2	− 0.6	0.4	1	0.2
Palpebral opening	− 1.4	0	0	1.2	− 0.6	− 0.4	− 2.2	− 0.4	0.2	0.2	0.2	− 0.6
Shed tears	0	0.2	0	0	0	0	0	0	0	0	0	0
Salivation	0	0	0	0	0	0	0	0	0	0	0	0
Respiratory rate	0	0	0	− 1.8	− 1.2	0	− 1	0	0	0.2	0.2	− 0.6
Heart rate	0	0	0	− 0.8	0	0	− 1	0	0	0.4	− 0.2	0
Piloerection	0	0	0	1.2	2	1.2	0	0	0	0.6	0.4	0.6
Temperature	− 2	− 2	− 0.6	− 2	− 1	− 0.6	− 2.4	− 2.8	− 1.8	− 2.2	− 0.4	− 0.2
Skin color	0	0	0	− 1	− 0.6	− 0.8	0	0	− 0.2	0	0	0

^a^Referred in [9]

#### General behavior

Grooming was suppressed by NPB-22 0.25 h after combustion. Marshmallow as the negative control also suppressed grooming; however, the suppression disappeared 1 h after inhalation. In contrast, NPB-22 suppressed grooming for 1 h. Adamantyl-THPINACA also suppressed grooming for 1 h; this suppression was stronger than that of NPB-22. CUMYL-4CN-B7AICA suppressed grooming only 0.25 h after combustion.

NPB-22 also suppressed verticalness, and this suppression disappeared after 1 h. The negative control also suppressed verticalness but only at 0.5 h. The strongest suppression of verticalness was achieved by Adamantyl-THPINACA (for 1 h). Meanwhile, CUMYL-4CN-B7AICA did not suppress verticalness.

In addition, Adamantyl-THPINACA promoted aggressiveness 0.25 h after combustion and pain response only 0.5 h after combustion and suppressed sound response for 0.5 h. Meanwhile, NPB-22, and CUMYL-4CN-B7AICA did not affect aggressiveness, pain response, or sound response.

#### Neurological behavior

NPB-22 suppressed spontaneous activity for 1 h after combustion; however, the suppression disappeared at 0.5 h temporarily. Regarding abnormal gait, the mice had difficulty walking caused by NPB-22, and the abnormal gait disappeared at 1 h. Regarding abnormal position, the mice maintained position difficulty caused by NPB-22, and this abnormal position also disappeared at 1 h. Muscle tone was also suppressed by NPB-22, and this suppression continued until 0.25 h. In mice that ingested the negative control, no changes were observed regarding these observation items, except for spontaneous activity, which was suppressed only at 0.25 h. Adamantyl-THPINACA suppressed spontaneous activity for 0.5 h. Regarding abnormal gait, the mice had difficulty walking caused by Adamantyl-THPINACA for 0.5 h, and regarding abnormal position, the mice maintained position difficulty caused by Adamantyl-THPINACA for 1 h. Muscle tone was strengthened by Adamantyl-THPINACA for 0.5 h. CUMYL-4CN-B7AICA affected only the position of the mice, and they maintained position difficulty only at 0.25 h.

In addition, Adamantyl-THPINACA suppressed grip strength only at 0.25 h; however, NPB-22 and CUMYL-4CN-B7AICA had no effect on grip strength.

#### Autonomic behavior

NPB-22 accelerated palpebral opening slightly 0.25 h after combustion. Conversely, the negative control closed the palpebrae of the mice at 0.25 h. Adamantyl-THPINACA closed the palpebrae of the mice more strongly than the negative control at 0.25 h. CUMYL-4CN-B7AICA did not affect the palpebral opening of the mice under study. The respiratory rate was suppressed by NPB-22 for 0.5 h. Adamantyl-THPINACA suppressed the respiratory rate only at 0.25 h. The negative control and CUMYL-4CN-B7AICA had no effect on the respiratory rate. NPB-22 strongly accelerated piloerection for 1 h, whereas the negative control, Adamantyl-THPINACA, and CUMYL-4CN-B7AICA did not induce piloerection. The temperature of the mice under study was lowered by NPB-22 for 0.5 h, a result equivalent to that of the negative control. Adamantyl-THPINACA lowered the temperature more strongly for 1 h than NPB-22. CUMYL-4CN-B7AICA lowered the temperature of the mice under study only at 0.25 h. NPB-22 changed the skin color of the mice to white only at 0.25 h. The negative control, Adamantyl-THPINACA, and CUMYL-4CN-B7AICA did not affect the skin color of the mice under study.

In addition, CUMYL-4CN-B7AICA increased the pupil size slightly only at 0.5 h, and Adamantyl-THPINACA suppressed the heart rate slightly for 0.25 h. NPB-22 and the negative control did not affect both observation items.

### Identification and quantification of the thermal decomposition products of NPB-22

**Fig. 2:**
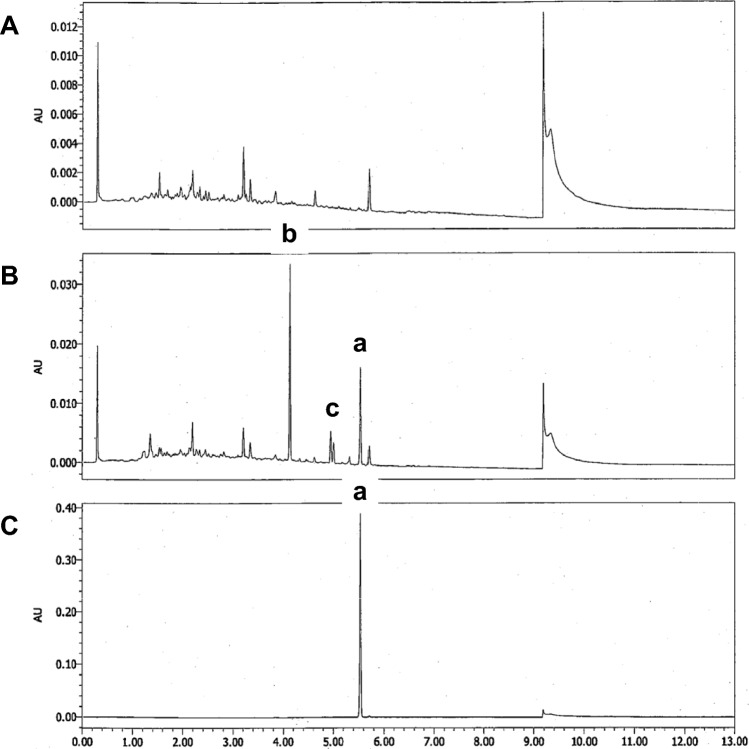
Chromatograms of the solution in the midget impingers with marshmallow as the negative control (**A**), NPB-22 (**B**), and the standard solution of NPB-22 (**C)**. Liquid chromatography is performed according to the method in the previous paper [8]

We obtained the smoke from the burned NPB-22, Adamantyl-THPINACA, and CUMYL-4CN-B7AICA using midget impingers during the inhalation exposure test and analyzed the samples using HPLC–PDA to determine the thermal decomposition products of these SCs. In the chromatogram of the burned NPB-22, two specific peaks were detected (peaks b and c in Fig. 2); therefore, thermal degradation of NPB-22 by combustion was recognized, and then we attempted to identify the degradation compounds of NPB-22. The chromatograms of the burned Adamantyl-THPINACA and CUMYL-4CN-B7AICA are shown in the Supplementary Material. A few peaks derived from the thermal degradation of CUMYL-4CN-B7AICA were observed, whereas those peaks were not detected in the chromatogram of Adamantyl-THPINACA.

**Fig. 3:**
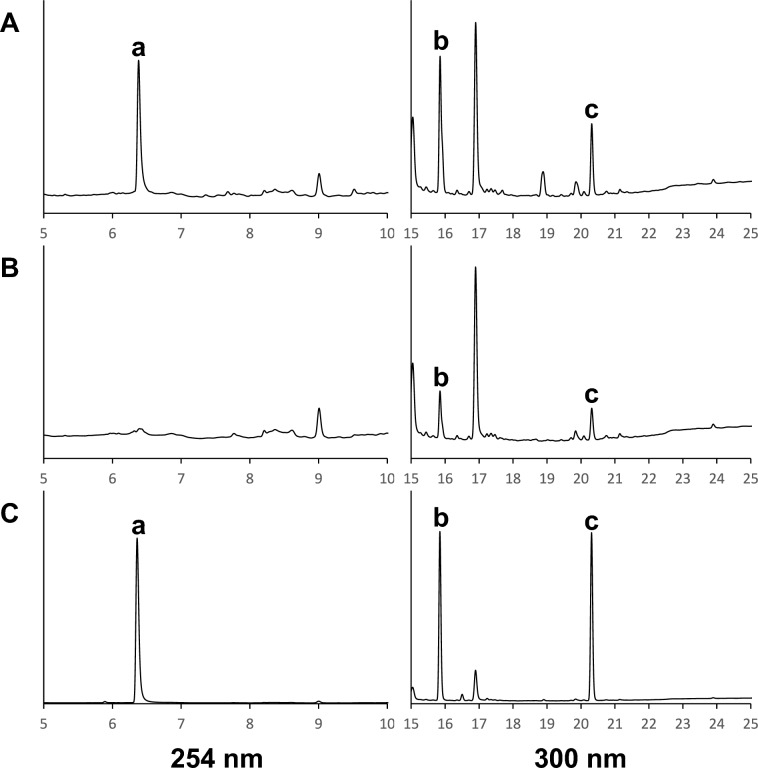
Chromatograms of the acetonitrile solution (**A**) and DMSO solution (**B**) in the midget impingers of NPB-22 and the mixed standard solution of NPB-22, 8-quinolinol, and pentyl indazole 3-carboxylic acid (**C**). The peaks are as follows: a, 8-quinolinol; b, pentyl indazole 3-carboxylic acid; c, NPB-22. Liquid chromatography is performed according to the method in this paper

PB-22, which is the indole analog of NPB-22, was reported to undergo thermolytic cleavage around the ester bonds [10]. Because NPB-22 also possesses an ester bond, we expected that NPB-22 hydrolyzes into 8-quinolinol and pentyl indazole 3-carboxylic acid on the ester bond. The solution in the midget impingers and the standard solutions of 8-quinolinol and pentyl indazole 3-carboxylic acid were analyzed using HPLC–PDA, and then the specific peaks of the midget impingers and standard solutions indicated a similar retention time (Fig. 3). In addition, the solutions in the midget impingers were mixed with each standard solution diluted to the same concentration as that in the midget impingers, and the mixed solutions were analyzed using HPLC–PDA. As a result, the peaks of any compounds were not split, and the two significant peaks of the solution in the midget impingers were identified—8-quinolinol and pentyl indazole 3-carboxylic acid. Therefore, it was revealed that NPB-22 was degraded to 8-quinolinol and pentyl indazole 3-carboxylic acid by combustion.

## Discussion

NPB-22 possessed CB_1_ activity equivalent to that of CP-55,940, the positive control, and Adamantyl-THPINACA. However, the influence of NPB-22 was weaker than that of Adamantyl-THPINACA in the inhalation exposure test. The smoke from the burned NPB-22 was analyzed, and it was revealed that NPB-22 was degraded to 8-quinolinol and pentyl indazole 3-carboxylic acid by combustion. Therefore, it was presumed that the actual amount of NPB-22 absorbed into the body was decreased by combustion, and its biological effects on the inhalation exposure test were weaker than the estimation on the [35S]GTPγS binding assays.

We prepared the calibration curves of NPB-22, 8-quinolinol, and pentyl indazole 3-carboxylic acid to calculate rate of thermal degradation of NPB-22. The correlation coefficients of the calibration curves of NPB-22, 8-quinolinol, and pentyl indazole 3-carboxylic acid were 0.99960, 0.99996, and 0.99997, respectively, in the range of 0.5–10 μg/mL; therefore, the calibration curves indicated satisfactory linearity. The molarity of NPB-22, 8-quinolinol, and pentyl indazole 3-carboxylic acid in the solutions of the midget impingers was calculated using the calibration curves. In the first midget impinger, the molarity of NPB-22, 8-quinolinol, and pentyl indazole 3-carboxylic acid was approximately 0.03, 0.05, and 0.08 mM, respectively, and in the second midget impinger, the molarity of the compounds under study was approximately 0.02, 0, and 0.03 mM, respectively. Incidentally, 8-quinolinol was not detected in the second midget impinger. Therefore, approximately 0.05, 0.05, and 0.11 μmol of NPB-22, 8-quinolinol, and pentyl indazole 3-carboxylic acid were recovered from the smoke of burned NPB-22. When NPB-22 degrades to 8-quinolinol and pentyl indazole 3-carboxylic acid, it is presumed that the thermal degradation compounds are generated in equal amounts. However, the amount of 8-quinolinol in the midget impingers was lower than that of pentyl indazole 3-carboxylic acid. Because it was presumed that 8-quinolinol was lost through the smoke pathway, the rate of thermal degradation of NPB-22 was estimated using the molarity of NPB-22 and pentyl indazole 3-carboxylic acid. As a result, it was estimated that 68% of NPB-22 was degraded by combustion. In addition, the degradation products of NPB-22, 8-quinolinol, and pentyl indazole 3-carboxylic acid did not possess CB_1_, and CB_2_ agonist activity. Therefore, absorbing NPB-22 like a cigarette was expected to affect CB_1_, and CB_2_ less than the results of in vitro assays, and the converted EC_50_ of the burned NPB-22 was found to be 1.56 × 10^−8^ and 8.47 × 10^−8^ mol/L for CB_1_ and CB_2_ respectively.

When the converted EC_50_ of the burned NPB-22 on CB_1_ was compared with the EC_50_ of Adamantyl-THPINACA and CUMYL-4CN-B7AICA, the order of them was Adamantyl-THPINACA > burned NPB-22 > CUMYL-4CN-B7AICA. CUMYL-4CN-B7AICA was also degraded by combustion; however, the amount of degraded CUMYL-4CN-B7AICA was of minimal quantity, and it was estimated that the thermal degradation of CUMYL-4CN-B7AICA did not affect the order of the converted EC_50_ on CB_1_. Grooming and verticalness were suppressed more strongly in this converted order on the inhalation exposure test. In addition, aggressiveness, sound response, and pain response were suppressed or promoted by Adamantyl-THPINACA temporarily; however, NPB-22 and CUMYL-4CN-B7AICA had no significant effects on these items. Adamantyl-THPINACA strongly lowered the body temperature of the mice under study. NPB-22 and CUMYL-4CN-B7AICA lowered it temporarily; however, this behavior was approximately equivalent to that of the negative control. The order of these items can also be explained by the converted order. Piloerection was observed only in mice that inhaled NPB-22. According to the reported results of a previous inhalation exposure test, Adamantyl-THPINACA and CUMYL-4CN-B7AICA, including other SCs (5F-MDMB-PICA, 5F-EMB-PINACA, AMB-CHMICA, 5F-MDMB-PINACA, THJ, and the herb products containing AB-CHMINACA and 5F-AMB) did not lead to piloerection [8, 13]. In addition, it has been reported that 8-quinolinol and pentyl indazole 3-carboxylic acid did not lead to piloerection; therefore, piloerection was specific to only NPB-22.

Drug abusers absorb SCs by inhaling the smoke from ignited cigarettes that are made by rolling the plant material in cigarette paper. It is reported that the ignition point of cigarettes reaches 950 °C, and some SCs undergo thermal degradation at the high temperature of cigarettes [9]. For example, the cyclopropyl ring in XLR-11 ([1-(5-fluoropentyl)-1*H*-indole-3-yl](2,2,3,3-tetramethylcyclopropan-1-yl)methanone) and UR-144 ((1-pentyl-1*H*-indole-3-yl)(2,2,3,3-tetramethylcyclopropan-1-yl)methanone) opens by thermal degradation; moreover, the thermal degradation compounds have been reported to possess higher CB_1_ activity than the parent compounds [10, 14]. In contrast to their SCs, the target compound of this study, NPB-22, was subjected to hydrolysis in its ester bond by thermal degradation, and the thermal degradation compounds did not possess CB_1_/CB_2_ activities.

Electronic cigarettes (e-cigarettes) are used recently by SC abusers [15, 16]. E-cigarettes heat some solutions to generate steam containing compounds (e.g., nicotine, and perfume). In fact, the solution that contains SCs has been reported to be sold in drug markets [17]. The range of heating temperature of e-cigarettes is 30–250 °C [18]. The degradation of NPB-22 was not observed at 250 °C with gas chromatography–mass spectrometry; therefore, when NPB-22 is absorbed using e-cigarettes whose heating temperature is 30–250 °C, it is not expected to undergo thermal degradation, and abusers could absorb more NPB-22 than smoking conventional cigarettes. Therefore, it is suggested that some SCs induce stronger biological effects when being absorbed using e-cigarettes even if they undergo thermal degradation using conventional cigarettes.

## Conclusion

NPB-22 had the same functional activity at cannabinoid receptors as Adamantyl-THPINACA. Meanwhile, the inhalation exposure tests showed that some biological effects of NPB-22 were weaker than those of Adamantyl-THPINACA. When analyzing the solution in the midget impingers recovering the smoke from the burned NPB-22 during the inhalation exposure test, approximately 70% of NPB-22 was degraded to 8-quinolinol and pentyl indazole 3-carboxylic acid by combustion. The results showed that the biological effects of NPB-22 were weakened by combustion. However, it has been recently reported that drug abusers absorb SCs using e-cigarettes. Because e-cigarettes heat solutions containing SCs at lower temperatures than conventional cigarettes, the compounds are expected not to undergo thermal degradation. Therefore, even if some new psychoactive substances are degraded thermally by conventional cigarettes and their biological activity is decreased, they could cause health hazards when using new devices for absorbing, such as e-cigarettes.

### Supplementary Information

Below is the link to the electronic supplementary material.

Supplementary file1 (PDF 486 KB)
