# Supplementary material for: The biological effects and thermal degradation of NPB-22, a synthetic cannabinoid
Source: Forensic Toxicol. 2024 Jan 31;42(2):142–51. doi: 10.1007/s11419-023-00679-5 (PMC11269510; doi:10.1007/s11419-023-00679-5)
Supplement: Supplementary file 1 — Supplementary file1 (PDF 486 KB) [file 11419_2023_679_MOESM1_ESM.pdf]

Supplementary material

Article title

The biological effects and thermal degradation of NPB-22, a synthetic cannabinoid

Journal name

Forensic toxicology

Author names

Akihiro Takeda[a*], Takahiro Doi[a], Akiko Asada[a], Katsuhiro Yuzawa[b], Akemichi Nagasawa[b], Kai Igarashi[c], Tomokazu Maeno[b], Atsuko Suzuki[b], Seiko Shimizu[b], Nozomi Uemura[b], Jun'ichi Nakajima[b], Toshinari Suzuki[b], Akiko Inomata[b] Takaomi Tagami[a]

[a]Division of hygienic chemistry, Osaka Institute of Public Health

1-3-3 Nakamichi, Higashinari-ku, Osaka 537-0025, Japan

[b]Department of Pharmaceutical and Environmental Sciences, Tokyo Metropolitan Institute of Public Health

3-24-1 Hyakunin-cho, Shinjuku-ku, Tokyo 169-0073, Japan

[c]Tolyo Metropolitan Island Public Health Center

2466-2 Okago, Hachijo-machi, Hachijojima, Tokyo 100-1492, Japan

*Corresponding author

Tel: +81-6-6972-1321

Fax: +81-6-6972-2393

E-mail: takeda@iph.osaka.jp

ORCID: 0000-0001-5368-7692

Chemical synthesis

Quinolin-8-yl 1-pentyl-1 H-indazole-3-carboxylate (NPB-22, compound ***7****)*

1-pentyl-1*H*-indazole-3-carboxylic acid was synthesized according to a previous paper [1]. This carboxylic acid (0.6 mmol) was dissolved in dichloromethane (12 mL). *N*, *N*-dimethylformamide (30 μL) and oxalyl chloride (100 μL) were added to a flask containing the carboxylic acid, which was stirred for 0.5 h. After the reaction, the solvent was removed under vacuum.

The residue was dissolved in dichloromethane, and 8-quinolinor (0.8 mmol) was added. Subsequently, triethylamine (360 μL) was added to the mixture, and the resulting solution was stirred at room temperature for 1 day. After the reaction, the solvent was removed under vacuum. Ethyl acetate and 0.1 mol/L $HCl_{(aq)}$ were added to the residue, and the solution was allowed to separate. The organic layer was washed with saturated aqueous NaCl and dried with anhydrous $MgSO_4$. The solution was filtered, and the solvent was removed under vacuum, and the residue was dissolved in dichloromethane. The solution was purified using an Isolela Speckt System on a SNAP KP-Sil (Biotage) with *n*-hexane/ethyl acetate (1:1, v/v). The solvent of the fraction containing the target product was removed under vacuum. The residue was dissolved in a small amount of dichloromethane, and the target product was precipitated by adding diethyl ether to yield compound **7** (0.2 mmol) as a white solid.

^{1}H NMR (DMSO-d_6): δ 8.87 (1H, dd, *J* = 4.0, 1.5 Hz), 8.50 (1H, dd, *J* = 8.5, 1.5 Hz), 8.15 (1H, d, *J* = 8.0 Hz), 8.00 (1H, dd, *J* = 8.0, 1.0 Hz), 7.94 (1H, d, *J* = 8.5 Hz), 7.77 (1H, dd, *J* = 8.0, 1.0 HZ), 7.71 (1H, t, *J* = 8.0 Hz), 7.62 (1H, dd, *J* = 8.5, 4.0 Hz), 7.56 (1H, t-like), 7.39 (1H, t-like), 4.62 (2H, t, *J* = 7.0 Hz), 1.95 (2H, s), 1.25-1.41 (4H, m, overlapped), 0.86 (3H, s). ^{13}C NMR (DMSO-d_6): δ 160.4, 150.7, 146.8, 140.6 (overlapped), 132.9, 136.3, 129.2, 126.9, 126.5, 126.3, 123.5, 122.4, 122.2, 121.9, 121.3, 111.0, 49.3, 29.1, 28.3, 21.7, 13.8.

1-(4-cyanobutyl)-N-(2-phenylpropan-2-yl)-1H-pyrrolo[2,3-b]pyridine-3-carboxamide
(CUMYL-4CN-B7AICA, compound ***9****)*

Oxalyl chloride (6 mmol) and N, N-dimethylformamide (0.1 mL) were added to compound **1** (5 mmol) in tetrahydrofuran (100 mL), and the solution was stirred for 0.5 h. The solvent was removed under vacuum. Methanol (60 mL) was added to the residue, and the solution was stirred for 4 h. The solvent was removed under vacuum. Ethyl acetate and purified water were added to the residue, and the solution was allowed to separate. The target product remaining in the water layer was transferred to an organic layer by adding sodium bicarbonate solution. The organic layer was dried with Na_2SO_4, the solution was filtered, and the solvent was removed under vacuum to yield compound **3** (2.8 mmol).

Potassium *tert*-butoxide (3.5 mmol) was added to compound **3** (2.8 mmol) in tetrahydrofuran (30 mL). After that, 5-bromovaleronitrile (21 mmol) was added, and the mixture was stirred at 40°C for 6 days. After the reaction, ethyl acetate, and purified water were added to the solution, which was allowed to separate. The organic layer was washed with saturated aqueous NaCl and dried with Na_2SO_4. The solution

was filtered, and the solvent was removed under vacuum. The residue was purified using an Isolela Speckt System on a SNAP KP-Sil (Biotage) with *n*-hexane/ethyl acetate (7:3 to 1:1, v/v). The solvent of the fraction containing the target product was removed under vacuum to yield compound **6** (1.5 mmol).

Tetrahydrofuran (8 mL), methanol (8 mL), and 1 mol/L $NaOH_{(aq)}$ (4 mL) were added to a flask containing compound **6** (1.5 mmol), and the mixture was stirred at room temperature for 4 days. After reaction, the organic solvent was removed under vacuum. Ethyl acetate and purified water were added to the remaining solution, and it was allowed to separate. The water layer was neutralized with 1 mol/L $HCl_{(aq)}$, combined with the organic layer, which was further separated from the water layer. The organic layer was washed with saturated aqueous NaCl and dried with Na_2SO_4. The solution was filtered, and the solvent was removed under vacuum.

Dichloromethane (30 mL), *N*, *N*-dimethylformamide (80 μL), and oxalyl chloride (7.5 mmol) were added to a flask containing the intermediate product (1.5 mmol), and the mixture was stirred at room temperature for 3 h. After the reaction, the solvent was removed under vacuum.

The residue was dissolved in dichloromethane (40 mL), and cumylamine (8.3 mmol) was added. Subsequently, triethylamine (1.2 mL) was added to the mixture, and the resulting solution was stirred at room temperature for 1 day. After the reaction, the solvent was removed under vacuum. Ethyl acetate and 1 mol/L $HCl_{(aq)}$ were added to the residue, and the solution was allowed to separate. The organic layer was washed with 0.05 mol/L $NaOH_{(aq)}$ and saturated aqueous NaCl and dried with Na_2SO_4. The solvent was removed under vacuum. The target product was precipitated by adding *n*-hexane/ethyl acetate to yield compound **9** (0.7 mmol) as a white solid.

^{1}H NMR (DMSO-d_6): δ 8.44 (1H, s), 8.33 (1H, dd, *J* = 8.5, 1.5 Hz), 8.29 (1H, dd, *J* = 4.5, 1.5 Hz), 8.01 (1H, brs), 7.41 (2H, d, *J* = 7.5 Hz), 7.28 (2H, t, *J* = 7.5 Hz), 7.14-7.18 (2H, t-like, overlapped), 4.34 (2H, t, *J* = 7.0 Hz), 2.57 (2H, t, *J* = 7.0 Hz), 1.95 (2H, m), 1.69 (6H, s), 1.58 (2H, m). ^{13}C NMR (DMSO-d_6): δ 163.0, 148.5, 147.1, 143.2, 130.9, 129.6, 127.8, 125.6, 124.7, 120.5, 119.0, 117.0, 109.0, 55.0, 43.3, 29.9, 28.8, 22.3, 15.8.

In vitro assays to evaluate CB_1 and CB_2 receptor activities

The reaction ratios of [^{35}S]-GTPγS binding of the compounds under study on CB1 and CB2 receptors are shown in Tables S1 and S2, respectively.

Table S1. Reaction ratio of [^{35}S]-GTPγS binding of the compounds under study on CB_1 receptor.

Substance	Substance concentration (mol/L)							
	1 × 10^{-12}	1 × 10^{-11}	1 × 10^{-10}	1 × 10^{-9}	1 × 10^{-8}	1 × 10^{-7}	1 × 10^{-6}	1 × 10^{-5}
	Reaction ratio (%)							
NPB-22	0.00	1.01	6.57	19.90	65.60	89.06	97.60	99.72
CP-55,940	1.56	0.73	8.41	33.13	88.03	100.00	99.21	100.00
Adamantyl-THPINACA	0.55	0.58	9.53	32.68	68.26	85.95	97.58	100.00
CP-55,940	0.00	0.00	2.85	29.69	77.58	97.62	95.34	96.94
CUMYL-4CN-B7AICA	0.00	0.00	1.11	3.51	20.95	67.17	100.00	100.00
CP-55,940	1.56	0.73	8.41	33.13	88.03	100.00	99.21	100.00
8-quinolinol	0.00	0.00	6.92	6.62	2.49	0.00	0.00	5.92
indazole 3-carboxylic acid	3.39	0.00	9.44	8.98	4.33	0.00	0.42	12.35
CP-55,940	2.11	11.28	24.78	53.67	78.35	92.98	96.31	98.49

Table S2. Reaction ratio of [^{35}S]-GTPγS binding of the compounds under study on CB_2 receptor.

Substance	Substance concentration (mol/L)							
	1×10^{-12}	1×10^{-11}	1×10^{-10}	1×10^{-9}	1×10^{-8}	1×10^{-7}	1×10^{-6}	1×10^{-5}
	Reaction ratio (%)							
NPB-22	0.00	0.26	0.80	9.18	37.84	68.94	88.54	95.99
CP-55,940	0.00	0.76	12.26	52.94	84.53	91.65	97.05	96.80
Adamantyl-THPINACA	0.00	0.00	0.00	3.44	1.21	4.27	9.43	29.89
CP-55,940	0.00	0.00	14.05	42.08	77.42	98.37	100.00	100.00
CUMYL-4CN-B7AICA	0.81	2.47	5.19	4.24	9.17	30.77	49.05	76.49
CP-55,940	0.00	0.76	12.26	52.94	84.53	91.65	97.05	96.80
8-quinolinol	0.00	0.00	0.66	2.84	0.00	0.00	0.00	0.00
indazole 3-carboxylic acid	0.00	0.00	13.94	9.26	5.57	0.00	0.00	6.60
CP-55,940	1.38	0.00	18.54	72.79	93.28	95.84	100.00	100.00

Confirmation of thermal degradation of Adamantyl-THPINACA and CUMYL-4CN-B7AICA

The equipment used for the inhalation exposure test was furnished with two midget impingers to recover the smoke from the burned SCs and to confirm whether the SCs underwent thermal degradation by analyzing the ingredients in the recovered smoke. The smoke was trapped using the first impinger, containing 20 mL of acetonitrile, and the second impinger, containing 20 mL of dimethyl sulfoxide, to collect crude SCs and their thermal degradation products before the smoke was exhausted from a safety cabinet (equipped with a HEPA filter). After the test, acetonitrile in the first impinger was concentrated under decompression in a water bath of 60°C, and dimethyl sulfoxide in the second bottle was concentrated using a vacuum vortex evaporator (BioChromato, Inc., Saitama, Japan) in a constant temperature of 40°C. Five mL of acetonitrile was accurately added to the residue after concentration, and the residue was dissolved by an ultrasonic wave. A part of the solution was filtered via a 0.2-μm filter and prepared as a sample solution for instrumental analyses.

Instrumental analyses were performed using a liquid chromatography system with a photo diode array detector (LC-PDA). LC-PDA analysis was performed using an ACQUITY UPLC PDA system (Waters). LC-PDA conditions were as follows: an ACQUITY UPLC HSS T3 column (particle size, 1.8 μm; 50 × 2.1 mm i.d.) (Waters) used at 40°C; mobile phase, (A) 0.1% formic acid aqueous, and (B) 0.1% formic acid acetonitrile solution at a flow rate of 0.6 mL/min. The gradient program was as follows: initially 99%A/1%B (0–0.5 min), linearly changed to 10%A/90%B (0.5–6.85 min), and then linearly changed to 1%A/99%B (6.85–8.85 min); injection volume, 1 μL. The wavelength of the ultraviolet (UV) spectra was 200–400 nm.

The chromatograms of the smoke extract from burned Adamantyl-THPINACA and CUMYL-4CN-B7AICA are shown in Figures S1 and S2, respectively. As a result, the crude Adamantyl-THPINACA, and CUMYL-4CN-B7AICA were detected in the recovered smoke.

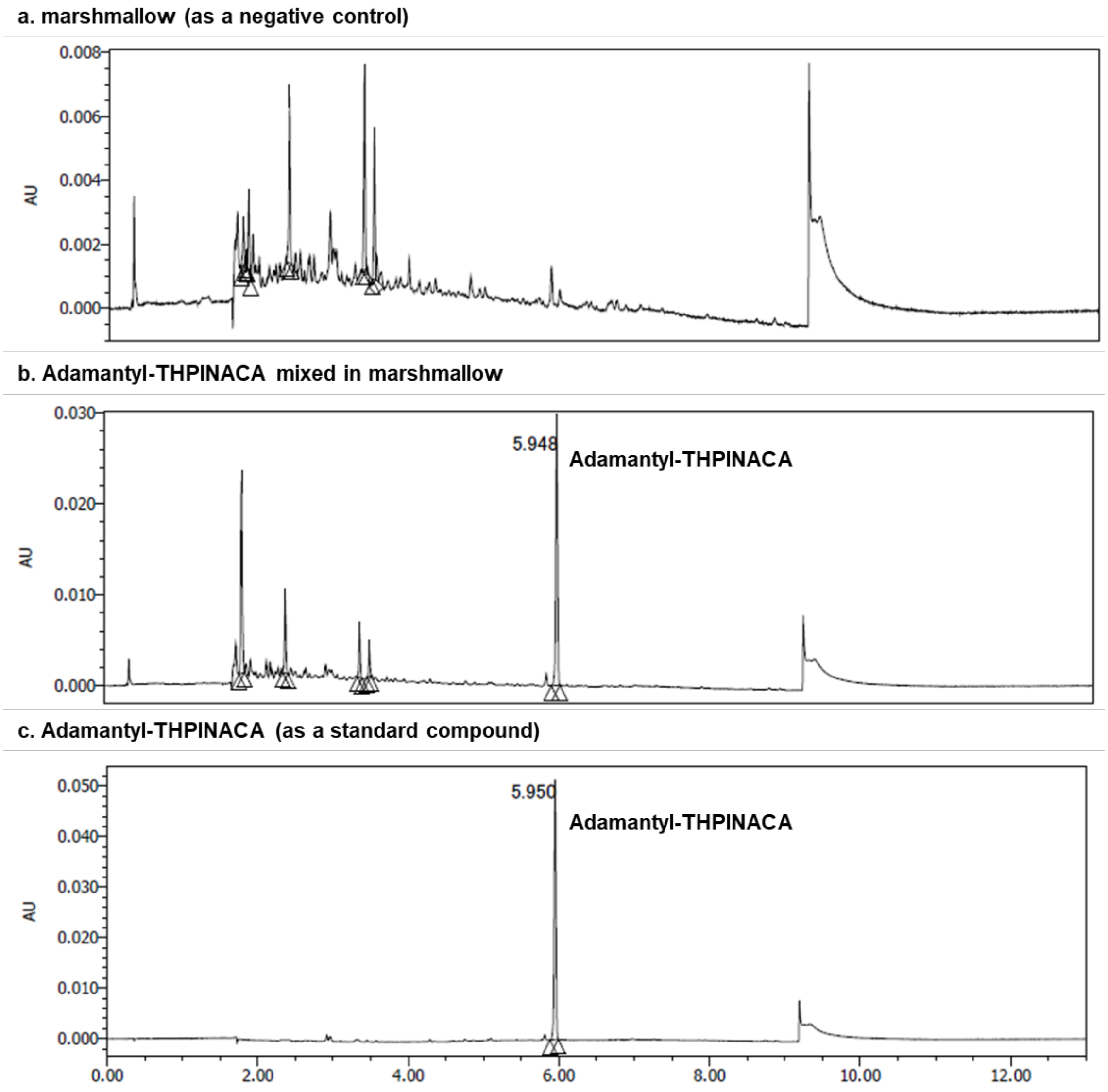

Figure S1. Chromatograms at 275 nm for the extract obtained from the recovered smoke from (a) burned marshmallow as the negative control, (b) burned Adamantyl-THPINACA mixed in marshmallow, and (c) Adamantyl-THPINACA solution for identifying its peak.

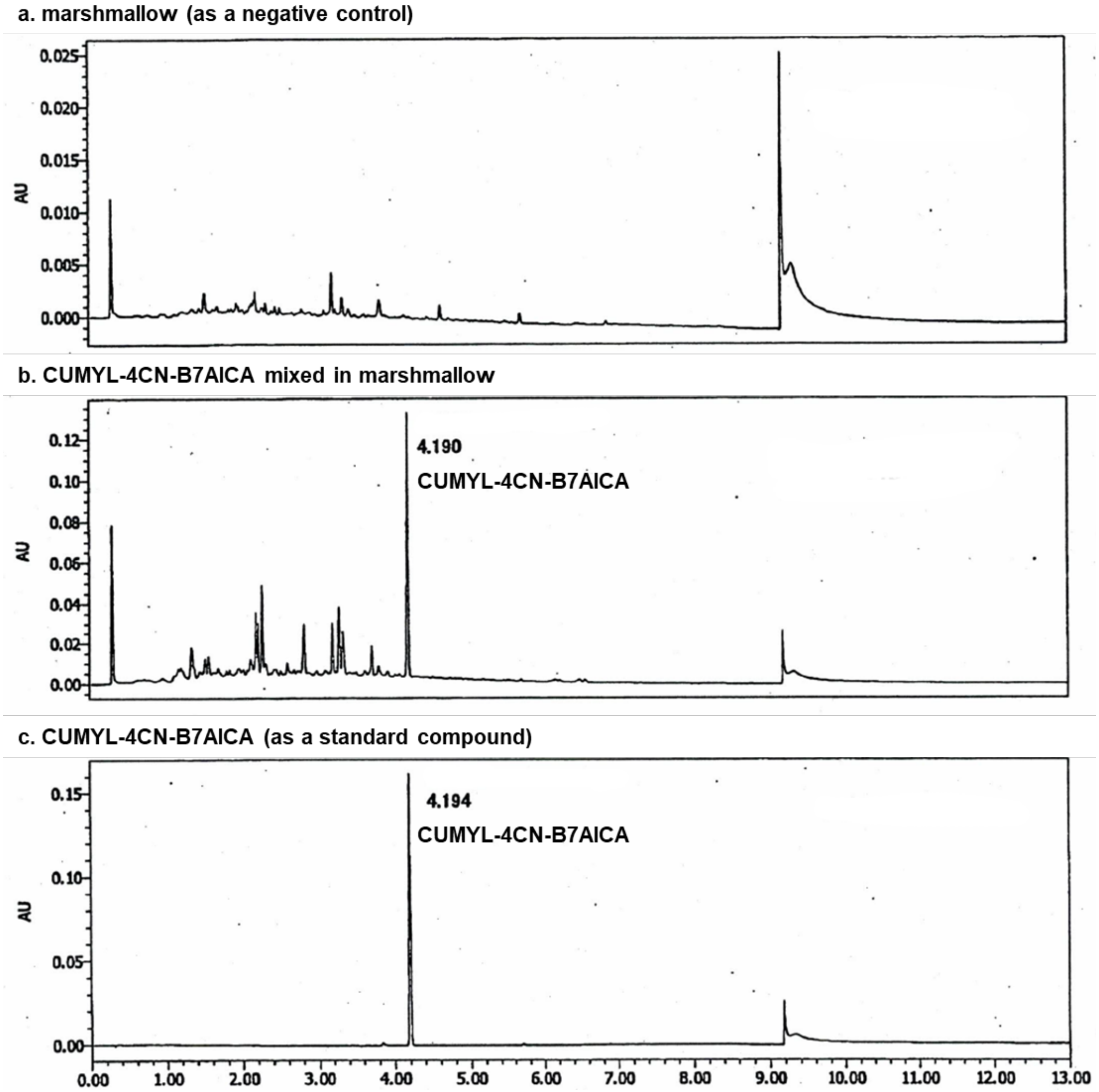

Figure S2. Chromatograms at 275 nm for the extract obtained from the recovered smoke from (a) burned marshmallow as the negative control, (b) burned CUMYL-4CN-B7AICA mixed in marshmallow, and (c) CUMYL-4CN-B7AICA solution for identifying its peak.

1. Asada A, Doi T, Tagami T, Takeda A, Sawabe Y (2017) Isomeric discrimination of synthetic cannabinoids by GC-EI-MS: 1-adamantyl and 2-adamantyl isomers of N-adamantyl carboxamides. Drug Test Anal 9:378–388. doi: 10.1002/dta.2124
